# Posttranslational regulatory mechanism of PD-L1 in cancers and associated opportunities for novel small-molecule therapeutics: PD-L1 posttranslational regulation and small-molecule therapeutics

**DOI:** 10.3724/abbs.2024085

**Published:** 2024-05-31

**Authors:** Minchen Cai, Mengting Xu, Dianping Yu, Qun Wang, Sanhong Liu

**Affiliations:** Shanghai Frontiers Science Center of TCM Chemical Biology Institute of Interdisciplinary Integrative Medicine Research Shanghai University of Traditional Chinese Medicine Shanghai 201203 China

**Keywords:** cancer, PD-L1, posttranslational modification, small molecule compound

## Abstract

Despite the tremendous progress in cancer research over the past few decades, effective therapeutic strategies are still urgently needed. Accumulating evidence suggests that immune checkpoints are the cause of tumor immune escape. PD-1/PD-L1 are among them. Posttranslational modification is the most critical step for protein function, and the regulation of PD-L1 by small molecules through posttranslational modification is highly valuable. In this review, we discuss the mechanisms of tumor cell immune escape and several posttranslational modifications associated with PD-L1 and describe examples in which small molecules can regulate PD-L1 through posttranslational modifications. Herein, we propose that the use of small molecule compounds that act by inhibiting PD-L1 through posttranslational modifications is a promising therapeutic approach with the potential to improve clinical outcomes for cancer patients.

## Introduction

Immunotherapy, which refers to the state of hypo- or hyperimmunity in the body, artificially enhances or suppresses the body’s immune function to achieve therapeutic disease treatment and is gradually becoming an important method for the treatment of cancer. Among numerous immunotherapeutic strategies, immune checkpoint blockade has shown clear advantages in the treatment of a range of cancers. Immune checkpoints increase antitumor immunity by blocking immune-related intrinsic downregulators, such as cytotoxic T lymphocyte antigen 4 (CTLA-4) and programmed cell death 1 (PD-1) or its ligand programmed cell death ligand 1 (PD-L1). Among them, PD-1 is an immune checkpoint receptor that restricts T-cell effector functions in tissues. It has also been the research focus of tumor immunotherapy in recent years, and it is widely used in the clinic. PD-L1, a known PD-1 receptor, can be expressed in normal cells when combined with PD-1 in immune cells to circumvent the killing of immune cells, thus effectively preventing autoimmune diseases, and is expressed in cancer cells to induce immunosuppression and thereby promote immune escape. Cancer immunotherapy is a therapeutic approach to reinitiate and maintain the tumor immune cycle and restore the body’s antitumor immune response so that immune cells can recognize and kill tumor cells normally, thereby achieving control and clearance of tumor cells in the body. Common tumor immunotherapies include immune checkpoint inhibitors and adoptive cell therapies.

**Figure 1:**
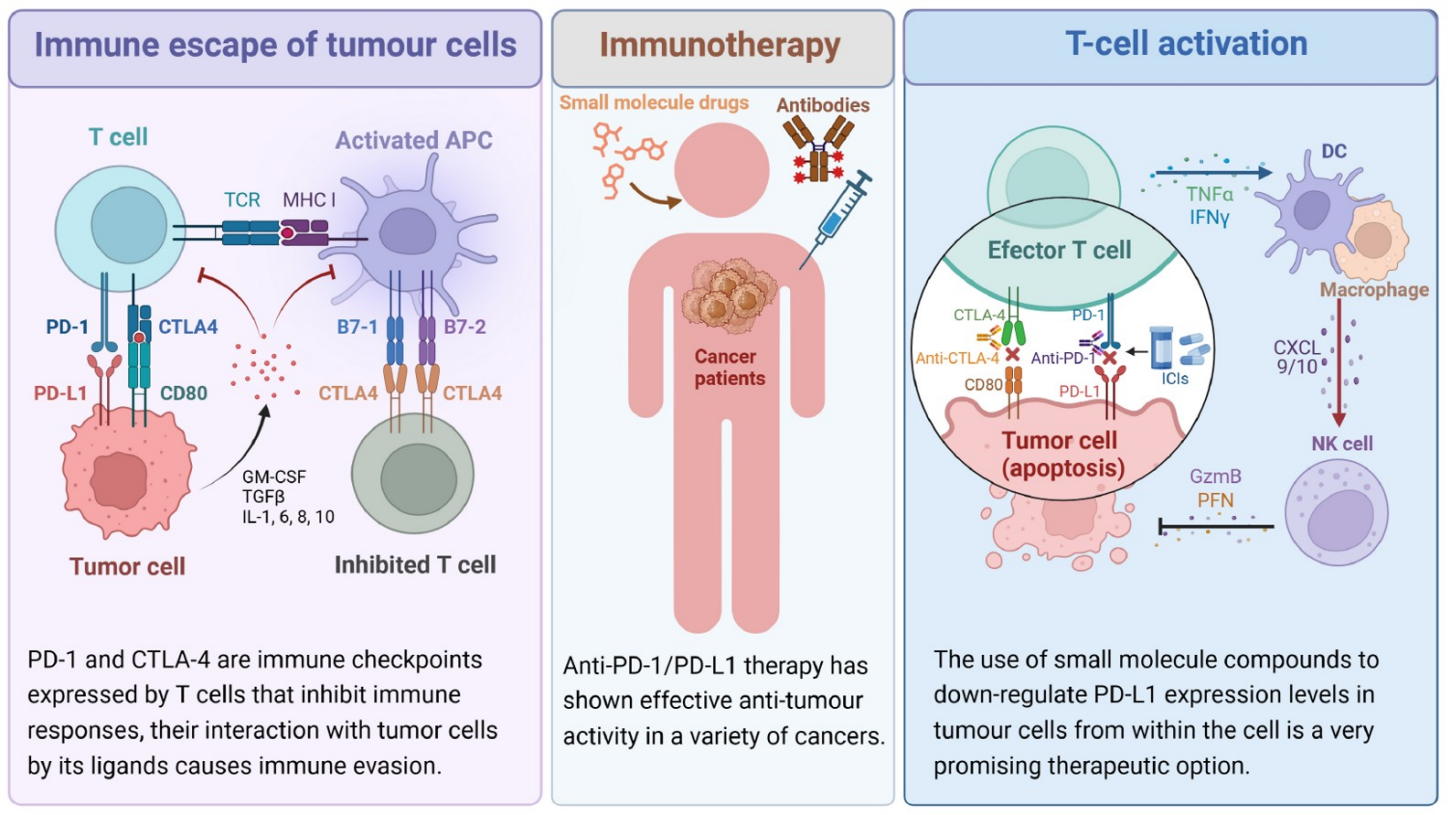
Figure 1 Mechanisms of immunotherapy Tumor cells inhibit the killing of tumors by immune cells through the upregulation of immune checkpoint molecular receptors, thus achieving immune escape. The classical immune checkpoints and their receptors are PD-1 and CTLA-4. The use of immune checkpoint inhibitors can prevent the binding of immune checkpoints to immune receptors and thus activate T cells to exert antitumor effects.

In 2014, the first PD-1 monoclonal antibody, nivolumab, was approved for the treatment of melanoma [ 1, 2] . To date, α-PD-1/PD-L1 therapy has shown potent antitumor activity in various cancers, such as melanoma, non-small cell lung cancer (NSCLC), gastric cancer, liver cancer, urothelial cancer, lymphoma, and all microsatellite instability (MSI)-high cancers. Immune checkpoint inhibitors (ICIs) have emerged as highly effective therapies for many cancers. All approved ICIs to date are mAbs, but mAbs, as a type of macromolecular therapeutic, suffer from many inherent drawbacks, including poor oral bioavailability, prolonged tissue retention and half-life, and poor membrane permeability, transport and storage. In addition, the higher cost of antibody drugs is also a nonnegligible issue. Therefore, downregulating the expression of PD-L1 in tumor cells using small molecule compounds is a very promising treatment option ( Figure 1).

PD-L1 within tumor cells is translated posttranslationally through the upper membrane but also undergoes a series of posttranslational modifications that stabilize the structure or promote degradation. The aim of reducing PD-L1 expression can be achieved both by inhibiting the transcriptional translation of PD-L1 to promote the reduced synthesis of PD-L1 and by modulating the posttranslational modification of PD-L1 to block or promote its stabilizing effect. Modulating the posttranslational modification of PD-L1 with small-molecule compounds to destabilize or promote its degradation, thereby enhancing the efficacy of antitumor immunity, holds great promise.

This review first describes the mechanisms underlying tumor escape mediated by PD-1/PD-L1 and briefly summarizes the limitations and effectiveness of anti-PD-1/PD-L1 mAbs and the need to develop small molecule drugs. Posttranslational modifications of PD-L1 are highlighted, and several small molecule compounds that undergo different posttranslational modifications to disrupt the PD-1/PD-L1 axis are exemplified.

## Immune Evasion Mechanisms via PD-1/PD-L1

Tumor immune escape is a mechanism by which tumor cells have evolved to escape killing by the body’s immune system, and tumor cells achieve immune escape by inhibiting tumor killing by immune cells through upregulation of immune checkpoint molecules or downregulation of their receptor molecules [3]. The classic immune checkpoint and its receptor, PD-1/PD-L1, play important roles in tumor immune escape. PD-1, also known as CD279 and PDCD1, is a type I transmembrane protein consisting of 288 amino acid residues and belongs to the B7/CD28 receptor superfamily [ 4, 5] . PD-1 has two ligands, PD-L1 (CD274) and PD-L2 (CD273), which also belong to the B7/CD28 family and consist of 290 and 270 amino acid residues, respectively; these proteins are type I transmembrane proteins, and PD-L1 and PD-L2 share 37% sequence homology. However, the expression range of PD-L2 was not as broad as that of PD-L1, leading to PD-L1 playing a more dominant role in tumor cell immune escape.

The protein structure of PD-1 can be divided into four parts: the immunoreceptor-based switch motif (ITSM), immunoreceptor tyrosine-based inhibitory motif (ITIM), immunoglobulin variable region (IGV), and transmembrane region [ 4– 8] . The interaction between PD-1 and PD-L1 can promote the phosphorylation of ITSM and ITIM in the intracellular domain of PD-1, which can recruit the tyrosine acid phosphatases Src homology phosphatase 1 (SHP-1) and Src homology phosphatase 2 (SHP-2) [ 8, 9] . These phosphatases dephosphorylate key proteins in the T-cell antigen receptor (TCR) signaling pathway and inhibit signaling pathway downstream of the TCR, such as protein kinase B (PKB/Akt), phosphoinositide 3-kinase (PI3K), mammalian target of rapamycin (mTOR), rat sarcoma (RAS), mitogen activated protein kinase (MAPK/MEK), and extracellular regulated protein kinase (ERK), and can inhibit the transcription of related genes, hindering the progression of the T-cell cycle and the expression of related proteins and ultimately inhibiting cytokine production and the proliferation and differentiation of T cells, leading to their loss of immune function and thus immune escape of tumor cells [ 10, 11] .

## Posttranslational Modification of PD-L1

PD-L1 expression within the cell is regulated at multiple molecular levels. PD-L1 transcription and mRNA stability are significantly regulated by oncogenic pathways, such as the c-myc, RAS, and epidermal growth factor receptor (EGFR) pathways [ 12– 15] . In addition, the PD-L1 protein undergoes various posttranslational modifications, including phosphorylation [ 16, 17] , glycosylation [ 16, 18, 19] , palmitoylation [ 20, 21] , acetylation [22], polyubiquitination [23], and deubiquitination [24], which can lead to subcellular translocation or changes in protein stability. Among these modifications, glycosylation, palmitoylation, acetylation and deubiquitination can increase the stability of the PD-L1 protein and prevent PD-L1 degradation, thus exerting normal effects on the membrane, while most phosphorylation and polyubiquitination occur by modifying PD-L1 degradation, preventing PD-L1 from functioning normally on the membrane. However, there are also minor moieties involved in phosphorylation and ubiquitination that can stabilize the structure of PD-L1.

### Glycosylation of PD-L1

N-chain glycosylation is a biosynthetic secretory pathway in the endoplasmic reticulum (ER) and Golgi apparatus [ 25, 26] . The N-linked glycosylation process is initiated in the endoplasmic reticulum by an oligosaccharyltransferase that transfers a 14 sugar core glycan from dolichol phosphate to an asparagine residue of the N-X-T/S motif in a newly synthesized nascent protein that has entered the endoplasmic reticulum lumen (where N is an asparagine, X is any amino acid except proline, S is a serine, and T is a threonine) [ 25, 26] . The decorin is then sheared and further processed in the endoplasmic reticulum and Golgi apparatus before the glycosylated protein is transported to the cell membrane [ 25, 26] . Once glycosylation is dysregulated, the protein is transported into the cytoplasm and rapidly undergoes ER-associated degradation [27]. A previous study showed that in most cancer cells expressing PD-L1, a high degree of PD-L1 glycosylation is detectable [16].

Although the 33 kDa form of nonglycosylated PD-L1 can be detected by western blot (WB) experiments, most PD-L1 was shown to be glycosylated, with molecular weights ranging from 45‒55 kDa [16]. The migration of PD-L1 on SDS-PAGE can be altered by glycosylation inhibitors of the N-chain but not the O-chain, suggesting that PD-L1 is predominantly N-glycosylated [18]. Mass spectrometric analysis revealed that the asparagine residues of the four N-X-T/S motifs in the extracellular domain (N35, N192, N200, and N219) of PD-L1 are heavily glycosylated. Replacement of these four asparagines by glutamine (4NQ mutant) abolished the glycosylation of PD-L1 and inhibited the migration of PD-L1, as shown by SDS-PAGE [16].

N-chain glycosylation of PD-L1 is important for PD-L1-mediated immunosuppression. Glycosylation may stabilize the protein structure of PD-L1. Li and coworkers demonstrated in cycloheximide chase experiments that the protein half-life of glycosylated PD-L1 was at least 4-fold longer than that of nonglycosylated PD-L1, suggesting that glycosylation of PD-L1 enhances its protein stability [16].

### Phosphorylation of PD-L1

PD-L1, which is not posttranslationally modified, is an unstable protein with a half-life of approximately 4 h and is approximately 33 kDa [28]. Li *et al*. [16] reported that nonglycosylated PD-L1 is labelled with ubiquitin after being phosphorylated by glycogen synthase kinase 3β (GSK 3β) and then degraded by the proteasome system. GSK-3β first phosphorylates PD-L1 at T180 and S184, while phosphorylated PD-L1 is labelled by the E3 ligase β-TrCP, ultimately leading to its degradation.

GSK3α may also mediate the phosphorylation of PD-L1 at the S279 and S283 sites in a similar manner, which in turn promotes reciprocal binding between PD-L1 and the E3 ubiquitin ligase ARIH1, thereby inducing the polyubiquitination of PD-L1 and its degradation through the proteasome pathway [29].

In addition to GSK3α/β, adenosine 5′-monophosphate (AMP)-activated protein kinase (AMPK) has also been confirmed to phosphorylate the S195 site of PD-L1, leading to its abnormal glycosylation and excessive accumulation in the endoplasmic reticulum for degradation [30]. PD-L1 can also undergo phosphorylation at S283, interfering with the interaction between PD-L1 and CMTM4, which leads to its degradation [31].

Different phosphorylation sites on PD-L1 promote PD-L1 degradation and stabilize its structure. For example, in pancreatic cancer, NEK2 phosphorylates the T194 and T210 sites of PD-L1, stimulates the glycosylation of PD-L1 at N192, N200, and N219, and prevents PD-L1 degradation [32]. IL-6-activated JAK1 phosphorylates the Y112 site of PD-L1, promoting the recruitment of STT3A to glycosylate PD-L1 to stabilize the structure of PD-L1 [17].

### Polyubiquitination of PD-L1

Accumulating evidence indicates that PD-L1 is regulated by the ubiquitin/proteasome pathway, suggesting that targeting PD-L1 polyubiquitination is an alternative approach to enhance immune checkpoint therapy [ 16, 23, 24, 33, 34] . Ubiquitination of proteins is a process coregulated by interactions between E2 ubiquitin conjugating enzymes, E3 ubiquitin protein ligases, and deubiquitinating enzymes (DUBs). The linkage between ubiquitin and a protein is first made by an E3 ubiquitin ligase, whereas a ubiquitin chain is tagged to a target protein and degraded [35].

The E3 ligase β-TrCP in basal-like breast cancer can phosphorylate the T180 and S184 residues of PD-L1 and enable it to undergo degradation [16]. Zhang *et al*. [23] reported that the E3 ubiquitin ligase SPOP is regulated and functions by cyclin D-CDK4, which is regulated by CDK4/6. Studies have shown that CDK4/6 increases the expression of PD-L1 by blocking cyclin D-CDK4-mediated phosphorylation of SPOP and subsequently promoting SPOP degradation. In EGFR-mutated non-small cell lung cancer, Qian *et al*. [36] identified a novel E3 ubiquitin ligase, MARCH8, which can mediate the degradation of PD-L1 induced by EGFR inhibition. After phosphorylation of PD-L1 at the S195 site mediated by AMPK, abnormal glycosylation is induced, preventing PD-L1 translocation from the ER to the Golgi, and PD-L1 strongly binds to ERAD-related components, leading to the accumulation of PD-L1 in the ER and subsequent degradation of PD-L1 by the ERAD-related E3 ligase HRD1 [30]. Endoplasmic reticulum-related degradation commonly refers to the unfolding or misfolding of proteins during spatial folding; these proteins accumulate in the endoplasmic reticulum and are subsequently cleared and degraded in the endoplasmic reticulum. ERAD plays two roles in cancer. On the one hand, it may endow cancer cells with the ability to tolerate toxic glycoprotein stress, thereby helping cancer cells. On the other hand, acute ER stress can also induce apoptosis [37]. The phosphorylation of S193 of PD-L1 caused by AMPK, the presence of a mannose-rich glycan structure and the occurrence of ER accumulation are characteristic of typical ERAD substrates and are ultimately labelled and degraded by the ERAD-related E3 ubiquitinase HRD1 [30]. The E3 ligase STUB1 (also known as CHIP) can inhibit CMTM4/6-mediated stabilization of the PD-L1 structure [ 33, 34] . ARIH1 has been identified as an E3 ubiquitin ligase, with GSK3α-mediated phosphorylation of PD-L1 at the S279 and S283 sites, and ARIH1 targets PD-L1 for further degradation. Jing *et al*. [38] reported that after FGFR3 is inhibited, obvious upregulation of PD-L1 expression is observed, while cancer cells with high FGFR3 expression exhibit low PD-L1 expression. They showed that activated FGFR3 can recruit and phosphorylate NEDD4, while phosphorylated NEDD4 has a greater ability to ubiquitinate it. NEDD4 then targets and catalyzes the K48-linked polyubiquitination of PD-L1 for PD-L1 degradation [38]. Wei *et al*. [39] reported that RNF125 is a new E3 ubiquitin ligase that can regulate PD-L1 ubiquitination and that RNF125 can bind to PD-L1 and negatively regulate PD-L1 expression through K48-linked polyubiquitination. Recently, Yang *et al*. [40] identified a new E3 ubiquitin ligase, ITCH, and identified its interaction with PD-L1. They found that the E3 ubiquitin ligase ITCH could bind to the K63 site of PD-L1 and promote PD-L1 polyubiquitination and then activate the lysosomal degradation pathway of PD-L1, which could reduce the drug resistance exhibited by PD-L1 upregulation caused by MAPK inhibitors; thus, ITCH overexpression could enhance the therapeutic effects of MAPK inhibitors [40].

However, not all ubiquitination is responsible for protein degradation. The K63 site of PD-L1 is thought to primarily control protein endocytosis, trafficking, and lysosomal degradation [41]. Li *et al*. [42] recently identified an E3 ubiquitin ligase, MIB2, whose ubiquitination at the K63 site of PD-L1 mediated by RAB8 is a sorting signal that is clearly required for PD-L1 protein translocation from the Golgi to the plasma membrane via exocytosis, which is the last step of PD-L1 execution outside before immune evasion. The ubiquitination of PD-L1 by MIB2 is a nonproteasomal posttranslational modification [42].

### Deubiquitination of PD-L1

Protein ubiquitination is dynamic, and three main enzymes are involved in this process, namely, those that add ubiquitin (ubiquitin conjugating enzymes) and those that remove ubiquitin (deubiquitinating enzymes), as well as ubiquitin protein ligases that regulate the linkage of ubiquitin to [35]. Deubiquitinases, which mainly remove ubiquitin from proteins that have been tagged by ubiquitination, shield the protein of interest from degradation after being tagged and thereby stabilize, exerting its supposed role.

In the late 1990s, two major families of deubiquitinating enzymes were identified, including UB carboxyl terminal hydrolases (UCHs) and UB-specific proteases (USPS/UBPs) [ 35, 43] . Deubiquitinating enzymes exhibit remarkable sequence diversity and thus may have broad substrate specificity [35]. As early as 2003, for example, Balakirev *et al*. [44] identified deubiquitinating activities for the novel deubiquitinases OTUB1 and OTUB2 of the outer protein superfamily. Later, Zhu *et al*. [45] discovered that the deubiquitinating enzyme OTUB1 can interact with and deubiquitinate PD-L1, which has undergone ubiquitination, thereby hindering its degradation via the endoplasmic reticulum-associated degradation pathway. Lim *et al*. [24] reported that CSN5 in the CSN family has deubiquitinating activity and is a novel deubiquitinating enzyme. They further demonstrated that the deubiquitinase CSN5 is essential for tumor-infiltrating macrophages to suppress antitumor immune responses by catalyzing proteolysis to remove polyubiquitination of PD-L1. The secretion of the proinflammatory factor TNF by macrophages-α activates NF-κB, which in turn induces CSN5 expression in cancer cells. Subsequently, CSN5 inhibits PD-L1 ubiquitination and degradation and enhances the PD-L1/PD-1 interaction, thereby facilitating escape from T-cell immune surveillance [24]. Wang *et al*. [46] demonstrated that USP22 removes the K6-, K11-, K27-, K29-, K33-, and K63-linked polyubiquitin chains of PD-L1 and reduces PD-L1 polyubiquitination, thus protecting PD-L1 from degradation by proteasomal recognition and increasing its stability, indicating that USP22 is a newly identified deubiquitinating enzyme. Xia *et al*. [47] identified a novel deubiquitinating enzyme, USP21, confirming that USP21 is a novel PD-L1-related deubiquitinase and that overexpression of USP21 significantly increases PD-L1 expression. Wu *et al*. [48] reported that USP9X in oral squamous cell carcinoma could induce the deubiquitination of PD-L1, reduce the polyubiquitination of PD-L1 and stabilize its structure, prompting cancer cells to undergo immune escape. Xu *et al*. [49] reported that USP20, a member of the USP family, can induce the deubiquitination of PD-L1, guaranteeing its stability. The lncRNA TINCR upregulates USP20 through the dual effect of ceRNA interaction and transcriptional repression of miR-199a-5p, thereby stabilizing the structure of PD-L1 by inhibiting its ubiquitination.

### Palmitoylation of PD-L1

Palmitoylation is a posttranslational modification of proteins, primarily by covalent attachment of palmitate to cysteine residues of proteins of interest via thioester linkages (also known as S-palmitoylation) [50]. Palmitoylation plays an important role in human physiological and pathological processes, including cancer, by affecting protein membrane anchorage, trafficking, interaction, and degradation [ 51, 52] . Lipid modification has also been shown to play an important role in the regulation of cell membrane proteins, and palmitoylation is an important and widely studied posttranslational lipid modification of proteins. For example, several cancer-related proteins, such as EZH2 [53], TEAD [54], and c-Met [55], are palmitoylated to stabilize their protein structure, and downregulation of the EZH2 palmitoyltransferase ZDHHC5 level significantly inhibits glioma tumor growth [53]. Yang and colleagues reported that PD-L1 could also be regulated by palmitoylation [21]. Palmitoylation plays an important role in regulating PD-L1 stability, and disrupting PD-L1 palmitoylation by site-specific point mutations or inhibiting the expression of its palmitoyltransferase ZDHHC9 can sensitize cancer cells to T-cell killing and in turn inhibits tumor growth [21]. Subsequently, Yao *et al*. [20] discovered another palmitoyltransferase, ZDHHC3, that regulates PD-L1 palmitoylation, and palmitoylation of PD-L1 by ZDHHC3 could inhibit the monoubiquitination of PD-L1, thus preventing the lysosomal degradation of PD-L1, leading to increased expression of PD-L1 and inhibiting the cytotoxic effect of T cells.

### Acetylation of PD-L1

Nonhistone lysine acetylation can compete with ubiquitination to affect protein stability or subcellular localization [ 56, 57] . Epidermal growth factor receptor (EGFR), which is mainly expressed on the plasma membrane, has been found to regulate clathrin-mediated endocytosis and nuclear localization through cyclic AMP response element binding protein (CBP) acetylation [ 58– 60] . In 2017, in an unbiased screen for posttranslational modifications, Horita *et al*. [61] first identified PD-L1 as a target of acetylation, but the specific mechanism involved was poorly defined. In 2020, Gao *et al*. [22] reported that PD-L1 is acetylated at the Lys263 site in the cytoplasmic domain by p300 acetyltransferase. Acetylation at the Lys263 site is a negative regulator of PD-L1 nuclear translocation, and nuclear PD-L1 binds to DNA and can regulate the expressions of multiple immune response-related genes and subsequently regulate antitumor immune responses [22]. Tumor migration has also been proven to be potentially related to the nuclear translocation of PD-L1, and nuclear PD-L1 also triggers the expressions of other immune checkpoint molecules that are not targeted by PD-1/PD-L1 blockade, leading to possible acquired immunotherapy resistance. Acetylation of PD-L1 can prevent the nuclear translocation of PD-L1, so promoting the acetylation of PD-L1 or directly preventing the nuclear translocation of PD-L1 can reduce acquired immunotherapy resistance [22].

## Small Molecule Compounds Regulate PD-L1 through Posttranslational Modification Pathways

To date, several PD-1/PD-L1-related mAbs, such as nivolumab, which is an anti-PD-1 antibody, and avelumab, which is an anti-PD-L1 antibody, have been marketed. However, when single monoclonal antibodies are administered, immune-related side effects are common, and due to their large molecular weight, poor permeability to tumor tissue after administration, and unsatisfactory treatment effect, most PD-1-related mAbs are available commercially via PD-L1 treatment of tumors. Therefore, the discovery of small molecule compounds that can posttranslationally modify PD-L1 by regulating it is of great interest for the clinical treatment of tumors.

### Regulating PD-L1 through glycosylation

N-glycosylation is a major posttranslational modification for stable structures of PD-L1 [16]. Inhibition of N-glycosylation of PD-L1 can decrease the stability of PD-L1, and PD-L1 can thus undergo degradation to prevent tumor immune escape due to high expression of PD-L1. Etoposide, for example, which was discovered by Hsu *et al*. [19], can be targeted by β-catenin to inhibit the expression of STT3, which is an N-oligosaccharyltransferase complex catalytic subunit; therefore, etoposide can inhibit the N-glycosylation of PD-L1, which in turn destabilizes the PD-L1 structure and causes its degradation. Shi *et al*. [62] discovered that TMUB1 enhances the N-glycosylation of PD-L1 by recruiting STT3A and subsequently enhancing its stability, thus promoting PD-L1 maturation and tumor immune evasion, and they also synthesized a competitive small-molecule polypeptide PTPR, which effectively inhibits the binding of PD-L1 to TMUB1, thereby exerting an antitumor effect by reducing the abundance of PD-L1 via N-glycosylation. BMS1166, a small molecule PD-L1 inhibitor developed by Bristol Myers Squibb, leads to the accumulation of PD-L1 in the ER by specifically affecting the glycosylation of PD-L1 and preventing the trafficking of underglycosylated PD-L1 from the ER. This finding illustrates that BMS1166 can decrease the expression of PD-L1 by inhibiting its glycosylation [63].

### Regulating PD-L1 through phosphorylation

Phosphorylation of PD-L1 is mainly regulated by GSK3α/β and AMPK, which can be rendered inactive by phosphorylation of unmodified PD-L1, accelerating degradation or blocking the remaining modifications. NEK2- and JAK-mediated phosphorylation of PD-L1 can promote its glycosylation to stabilize PD-L1.

ES-072 is an EGFR inhibitor. Wu *et al*. [29] reported that it can induce GSK3α activation, promoting the phosphorylation of the S279 and S283 sites of PD-L1, which in turn promotes the recruitment of the E3 ubiquitin ligase ARIH1 for the final degradation of PD-L1 via the proteasome pathway. Gefitinib is also an EGFR inhibitor. Studies have shown that IL-6 can activate JAK and then induce phosphorylation at the Y112 site of PD-L1, promoting the recruitment of STT3A to glycosylate PD-L1. Gefitinib can reduce the release of IL-6 by inhibiting EGFR, thus inhibiting PD-L1 phosphorylation and further decreasing glycosylated PD-L1, reducing PD-L1 abundance [12]. NCL00017509 is a NEK2 inhibitor. Zhang *et al*. [32] reported that NCL00017509 can inhibit the NEK2-induced phosphorylation of PD-L1 at the T194 and T210 sites by inhibiting NEK2, thus preventing PD-L1 glycosylation at the N192, N200, and N219 sites and subsequently accelerating PD-L1 degradation. Previous studies have shown that metformin can also promote the degradation of PD-L1. Metformin directly induces the phosphorylation of the S195 site of PD-L1 by activating AMPK, and the phosphorylation that occurs at the S195 site leads to abnormal glycosylation of PD-L1, failure to normally reach the membrane and thus excessive accumulation in the endoplasmic reticulum, resulting in the occurrence of endoplasmic reticulum-associated degradation [30].

### Regulating PD-L1 through polyubiquitination

Polyubiquitination, one of the major degradation pathways of PD-L1, is mainly characterized by the linking of ubiquitin chains together with PD-L1 via E3 ubiquitin ligases, which are then degraded by the proteasome via a recognition mark. Targeting ubiquitin ligases and their upstream proteins or genes with small molecules to promote PD-L1 degradation is a research hotspot. Partial EGFR inhibitors such as osimertinib promote the interaction of the E3 ubiquitin ligase MARCH8 with and polyubiquitination of PD-L1 for degradation by the proteasome pathway by inhibiting EGFR in EGFR-mutated non-small cell lung cancer [36]. Resveratrol, ciglitazone and troglitazone were found to induce E3 ubiquitin ligases in triple-negative breast cancer cells with β-TrCP expression, thereby decreasing PD-L1 protein expression in cancer cells [16]. Zhang *et al*. [64] reported that sulforaphane can directly induce the expression of the E3 ubiquitin ligase β-TrCP in cancer cells, thus leading to further activation of ubiquitination-mediated PD-L1 proteolysis, which in turn induces PD-L1 degradation. Yang *et al*. [65] identified the classical hypoglycemic drug canagliflozin as an inhibitor of SGLT2 that can disrupt the interaction between SGLT2 and PD-L1 and thus promote the interaction between PD-L1 and the E3 ubiquitin ligase SPOP, enabling PD-L1 degradation via the polyubiquitination proteasome pathway.

### Regulating PD-L1 through deubiquitination

Deubiquitination is a posttranslational modification that involves the removal of polyubiquitin chains from proteins labelled with ubiquitin chains by a deubiquitination enzyme, which can stabilize the target protein and exert corresponding effects. Small molecule compounds can reduce PD-L1 expression by inhibiting the activity of deubiquitinase. Research has shown that curcumin can inhibit the activity of the ubiquitination enzyme CSN5, induce the normal ubiquitination of PD-L1, and further promote its recognition and degradation by proteasomes, thereby inhibiting PD-L1-based immune escape, which is beneficial for the anti-CTLA-4 treatment of TNBC, melanoma, and colon cancer syngeneic mouse models [24]. Berberine (BBR), a common anti-inflammatory drug, has been found to specifically bind to the Glu76 site of CSN5, thereby inhibiting the deubiquitination activity of CSN5 in non-small cell lung cancer, reducing the expression of PD-L1, and promoting antitumor immunity [66].

### Regulating PD-L1 through palmitoylation

Palmitoylation is a lipid modification mainly catalyzed by palmitoyl transferase. The palmitoylation of PD-L1 is mainly mediated by two types of palmitoyltransferases, ZDHHC3 and ZDHHC9, both of which are members of the ZDHHC family. Currently, 2-bromo palmitate, a panpalmitoylation inhibitor that can inhibit the palmitoylation of PD-L1, which causes it to be labelled with monoubiquitin and then degraded through the lysosomal pathway, thereby enhancing antitumor immunity, is a small molecule compound that inhibits palmitoylation [20]. Wang *et al*. [67] demonstrated that benzosceptrin C can block the palmitoylation of PD-L1 by inhibiting the activity of DHHC3, thus promoting the lysosomal degradation of PD-L1, suggesting that benzosceptrin C could block the direct interaction between PD-L1 and PD-1 and exert an immune antitumor effect.

## Conclusion

**Figure 2:**
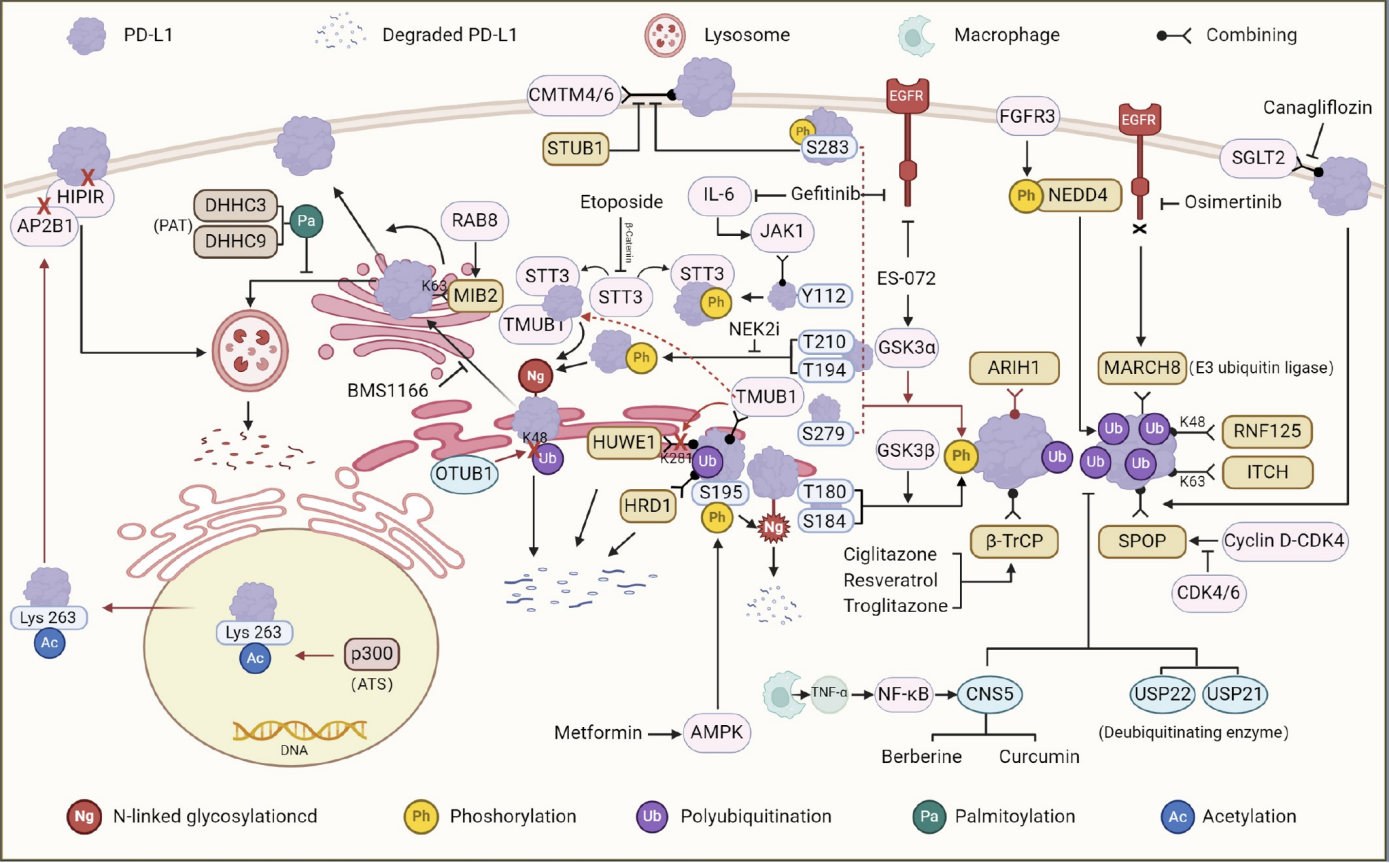
Figure 2 Posttranslational modifications of PD-L1 in tumor cells The intracellular PD-L1 protein is subjected to several forms of posttranslational modifications before membrane uploading, among which phosphorylation and polyubiquitination promote the degradation of PD-L1, whereas glycosylation, palmitoylation, acetylation, and deubiquitination can improve the stability of the PD-L1 protein to make it function normally on the membrane.

This review mainly discusses the posttranslational modifications related to PD-L1, as well as the degradation of PD-L1 by small molecule compounds by activating or inhibiting its posttranslational modifications ( Figure 2 and Table 1).

**Table:** **Table 1** Small molecule compounds shown to modulate PD-L1 in cancer cells by preclinical studies

Chemical compound	Targeted regulation of PTM	Effects of chemical compound on PD-L1	Ref
Etoposide, PTPR	Glycosylation	Etoposide can be targeted β-catenin to inhibit N-glycosylation of PD-L1 and promote its degradation. PTPR effectively inhibits the binding of PD-L1 to TMUB1, reducing the abundance of PD-L1 via N-glycosylation.	[ 19, 62]
BMS1166	Glycosylation	Leading to the accumulation of PD-L1 in the endoplasmic reticulum by specifically affecting the glycosylation of PD-L1.	[63]
ES-072, gefitinib	Phosphorylation	ES-072 can induce GSK3α activation, prompting phosphorylation of the S279 and S283 sites of PD-L1. Gefitinib can reduce the release of IL-6, thus inhibiting PD-L1 phosphorylation and further decreasing glycosylated PD-L1, reducing PD-L1 abundance.	[29, 12]
NCL00017509, metformin	Phosphorylation	NCL00017509 can inhibit NEK2-induced phosphorylation of PD-L1 at the T194 and T210 sites by inhibiting NEK2. Metformin directly induces the phosphorylation of the S195 site of PD-L1 by activating AMPK.	[32, 30]
Osimertinib, *etc*.	Polyubiquitination	Osimertinib promotes the E3 ubiquitin ligase MARCH8 to interact with and polyubiquitinates PD-L1 for degradation.	[36]
Resveratrol, ciglitazone, troglitazone, sulforaphane	Polyubiquitination	Resveratrol, ciglitazone and troglitazone induce E3 ubiquitin ligases in triple-negative breast cancer cells with β-TrCP expression. Sulforaphane can directly induce E3 ubiquitin ligase in cancer cells β-TrCP expression, which in turn induces PD-L1 to undergo degradation.	[16, 64]
Canagliflozin	Polyubiquitination	Canagliflozin can disrupt the interaction between SGLT2 and PD-L1 and thus promote the interaction between PD-L1 and the E3 ubiquitin ligase SPOP.	[65]
Curcumin, berberine	Deubiquitination	Curcumin can inhibit the activity of the ubiquitination enzyme CSN5, induce the normal ubiquitination of PD-L1. Berberine specifically bind to the Glu76 site of CSN5, thereby inhibiting the deubiquitination activity of CSN5 in non-small cell lung cancer.	[24, 66]
2-Bromo palmitate, benzosceptrin C	Palmitoylation	2-Bromo palmitate can inhibit the palmitoylation of PD-L1. Benzosceptrin C can block the palmitoylation of PD-L1 by inhibiting the activity of DHHC3, thus promoting the lysosomal degradation of PD-L1.	[20, 67]

PD-L1, a popular research subject in antitumor immunity, has also been developed as a new drug in recent years. The posttranslational modification of PD-L1 is influenced by small molecule compounds, further destabilizing its structure and degrading it, thereby disrupting the PD-1/PD-L1 axis to activate antitumor immunity.

There are six main PD-L1-related posttranslational modifications: glycosylation, phosphorylation, polyubiquitination, deubiquitination, palmitoylation and acetylation. Among these pathways are glycosylation, palmitoylation, deubiquitination and NEK2-mediated phosphorylation at T194 and T210 and JAK1-mediated phosphorylation at Y112. While degradation promotes sumoylation, GSK3β is mediated by the T180 and S184 sites, GSK3α mediates the phosphorylation of the S279 and S283 sites, and AMPK mediates the phosphorylation of the S195 and S283 sites. In addition to inhibiting the acetylation of nuclear PD-L1, nuclear PD-L1 binds to DNA, regulates the expression of multiple immune response-related genes, and regulates antitumor immune responses.

Although all of the approved marketed monoclonal antibodies, such as nivolumab and avilumab, are available, there are many reported small molecule compounds that regulate PD-L1 expression through the posttranslational modification pathway of PD-L1. Most small molecule compounds that regulate PD-L1 by glycosylation modulate its glycosylation via the catalytic subunit of the N-glycosyltransferase complex (STT3); for example, etoposide inhibits PD-L1 glycosylation by inhibiting STT3 expression, and the synthetic small molecule peptide PTPR, which targets tmub1, inhibits PD-L1 glycosylation by inhibiting its binding to PD-L1 and its ability to recruit STT3A. Small molecules that regulate PD-L1 expression through the phosphorylation of PD-L1, are mostly EGFR inhibitors. Among these agents, gefitinib, a representative partial EGFR inhibitor, can reduce the release of IL-6 by inhibiting EGFR, thus reducing the phosphorylation of PD-L1 by JAK, decreasing glycosylated PD-L1, and ultimately reducing PD-L1 abundance. There are also several EGFR inhibitors that affect PD-L1 phosphorylation through other pathways, such as ES-072, by inducing GSK3α activation to phosphorylate the PD-L1 protein, ultimately degrading PD-L1 through the proteasome pathway, whereas metformin can directly phosphorylate PD-L1 via AMPK, leading to its abnormal glycosylation and ultimately to degradation. Natural small molecule compounds play a prominent role in regulating ubiquitination and deubiquitinating modifications. Resveratrol and sulforaphane induce canonical E3 ubiquitin ligases by inducing β-TrCP, promoting its polyubiquitination and, in turn, the degradation of PD-L1 via the proteasome pathway. However, curcumin and berberine enhance antitumor immunity by inhibiting the activity of the deubiquitinase CSN5, which can accelerate the polyubiquitination and degradation of PD-L1. The number of studies on the palmitoylation and acetylation of PD-L1 has increased over the last few years, and there are few small molecule compounds that modulate these two modifications. Among them, small-molecule compounds that regulate palmitoylation currently include only the classical panpalmitoylation inhibitors 2-bromopalmitate and benzosceptrin C. Although there are few studies on PD-L1 acetylation, there are no reports on small-molecule compounds that regulate the expression of PD-L1 through acetylation.

Although monoclonal antibodies have many shortcomings, they are still the main treatment used in current cancer immunotherapy. Common immune checkpoint mAbs are mainly divided into anti-PD-1 mAbs, anti-PD-L1 mAbs and anti-CTLA-4 mAbs. In this review, we mainly discuss small molecule compounds that reduce the expression of PD-L1 in tumor cells through posttranslational modification and block the PD-1/PD-L1 axis from the root to promote immune escape. Therefore, is it possible to combine the small molecules listed in this review with anti-CTLA-4 mAbs? The study of Wang *et al*. [67] provided favorable evidence for our hypothesis. They reported that the natural small molecule benzosceptrin C can inhibit the palmitoylation of PD-L1 and accelerate its degradation by inhibiting the palmitoyl transferase dhhc3. This study proposed and verified the possibility of combining Benzosceptrin C with an anti-CTLA-4 mAb and revealed that the effect of combining Benzosceptrin C with an anti-CTLA-4 mAb was greater than that of using either agent alone, which plays a two-pronged role [67]. Therefore, the combination of small molecules that downregulate the expression of PD-L1 and monoclonal antibodies has good development and application prospects.

As an increasing number of studies on PD-L1 have been conducted, an increasing number of in-depth proteins that regulate the effect of PD-L1 posttranslational modification by interacting with PD-L1 are being discovered. Although there are only a few posttranslational modifications of PD-L1 and the structure of PD-L1 has been studied for a long time, the study of other proteins in human body is far more than that, and many proteins that may interact with PD-L1 have not been identified. For example, for the ubiquitination of PD-L1, in the last few months, two new E3 ubiquitin ligases, ITCH and MIB2, were discovered to interact with PD-L1, promote its degradation or stabilize its structure. The ubiquitination of proteins has been studied for decades, the number of E3 ubiquitin ligases that have been discovered is overwhelming, and very few are currently known to regulate PD-L1. Similarly, for deubiquitinases, many members of the USP family have not been found to be involved in the ubiquitination of PD-L1. Only two palmitoyltransferases, ZDHHC3/9, are currently known to regulate PD-L1 palmitoylation, and the remaining ZDHHC family members have not been found to regulate PD-L1 palmitoylation.

Other directions of posttranslational modification of PD-L1 should be explored. In other words, in addition to the six known posttranslational modifications related to PD-L1, new posttranslational modifications related to PD-L1 have been identified. Indeed, there are also c-/o-glycosylation, S-nitrosylation, methylation, and several other lipidation modifications in addition to S-palmitoylation, namely, phosphatidylation, myristoylation, and prenylation. Although no study has yet shown that PD-L1 has new posttranslational modifications, cells and proteins are far more complex than we thought. We cannot be sure that there are only six posttranslational modifications of PD-L1; rather, there are still undiscovered posttranslational modifications related to PD-L1, such as N-myristoylation. Myristoyl is a 14-carbon saturated fatty acid that can enhance the affinity of proteins for membranes. As a protein that needs to function on the membrane, PD-L1 may undergo N-myristoylation to help it function on the membrane. S-prenylation is also a lipidation modification. The target protein is modified by farnesyltransferase or geranylgeranyltransferase catalysis, which is called farnesylation or geranylgeranylation and is collectively known as prenylation. Prenylation can not only increase the affinity of proteins for the membrane but also enhance their hydrolytic stability. Therefore, PD-L1 is likely to undergo prenylation to increase its stability and subsequently function on the membrane. Moreover, if new posttranslational modifications are found, additional proteins or pathways regulate PD-L1, and additional small molecule compounds can be found to regulate PD-L1 through new posttranslational modifications. For example, once N-myristoylation is confirmed to be a PD-L1-related posttranslational modification, inhibitors of myristoyl transferase can be used as compounds to regulate PD-L1 for further preclinical research.

Similarly, with extensive and in-depth research, more compounds have been found to regulate PD-L1 through posttranslational modification. In particular, small molecule compounds have become a relevant research hotspot for PD-L1 because of their superior characteristics over monoclonal antibodies. Regulation by small molecule compounds is substantial, and two aspects can be illustrated. On the one hand, every year, many new proteins that regulate PD-L1 by interacting with PD-L1 have been discovered, which may be new targets for small molecules to regulate PD-L1. The compounds are generally multitargeted, which is the theoretical basis for the new use of old drugs. For example, the classical antimicrobial agent berberine, hypoglycemic agent canagliflozin, and others were found to regulate PD-L1 through posttranslational modification pathways. On the other hand, the vast majority of the sources of small molecule compounds are natural products, and many of their active ingredients are still undefined, especially traditional Chinese medicine (TCM) in China, which has clear drug efficacy, but they are not yet specific to small molecule monomeric compounds, which remain to be developed. The discovered monomeric small compounds can also be structurally modified by chemical reactions to achieve better efficacy. Although the ocean is a great treasure house for humanity, there are comparatively few relevant studies of natural products in the ocean, and there are still many natural products and their effective small molecular monomers waiting to be discovered. Both of these aspects illustrate the future prospects for new small molecule compounds that modulate PD-L1.

**Figure 3:**
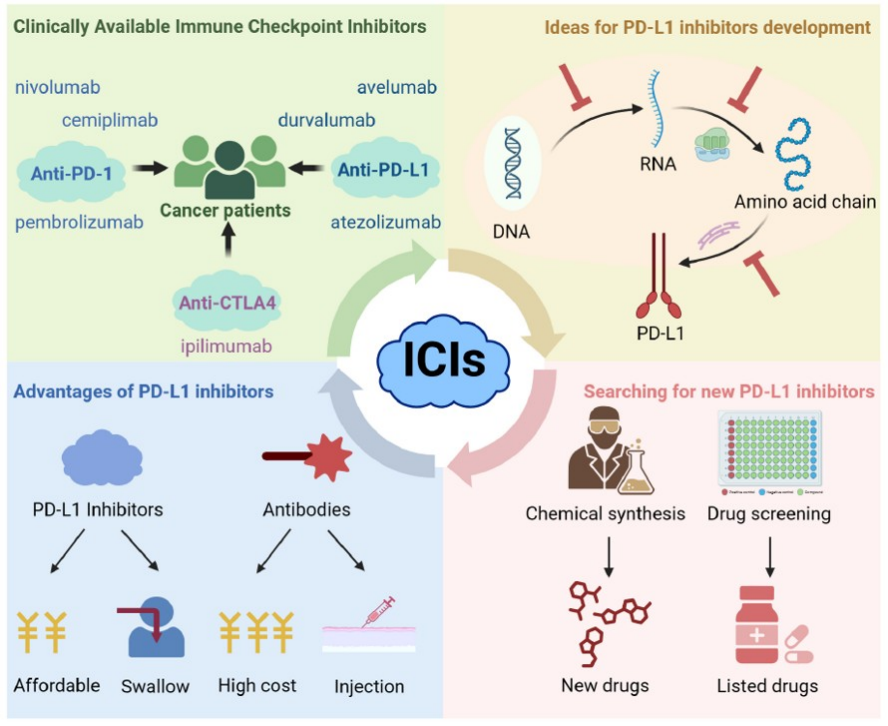
Figure 3 The search for new immune checkpoint inhibitors is promising Although CTLA-4 and PD-1/PD-L1 inhibitors have demonstrated significant efficacy, they have low response rates in most patients and are expensive; therefore, the development of new ICIs for cancer treatment is currently a major research interest.

There are many ways to affect PD-L1 expression in cells, and a small molecule compound may only affect PD-L1 expression in one regulatory way, which also leads to the possibility that the compound may only be effective on this type of tumor cell; therefore, in future clinical applications, there are certain limitations. Some EGFR inhibitors, such as osimertinib, can only inhibit PD-L1/2 in EGFR-mutated non-small cell lung cancer. Therefore, the search for small molecule compounds that can broadly regulate PD-L1 expression is one of the major future research directions in this field ( Figure 3).

Certainly, small-molecule compounds are not all advantages. Small molecule compounds have some unknown side effects. For example, small molecule compounds generally have multitarget effects, while studies generally focus on only one of them. For example, metformin, a classic hypoglycemic drug, was found to be an antitumor drug, and berberine, which was initially widely used as an antibacterial drug, was subsequently found to have lipid-lowering, antitumor and other effects. Whether these multitarget small molecule drugs still have undiscovered targets of action is uncertain. Among the undiscovered targets, they may also destroy normal cells. Moreover, marketed drugs have several side effects, such as occasional vomiting of metformin, abdominal pain and other adverse reactions, and berberine may be fatal if the side effects are serious. However, as researchers, it is our responsibility to determine the underlying mechanism and avoid these side effects so that patients can use these drugs for treatment without concern and avoid the pain of illness.

However, ultimately, new targets or new small-molecule compounds are to be discovered, with the intent of being applied in the clinic to treat patients afflicted by cancers, while the human *in vivo* environment is complex, and it is not the end point to find a small molecule compound that regulates PD-L1. As Professor Mien-Chie Hung stated, it is important to remember that some mechanisms depend on the cellular context and that appropriate combinations of biomarkers and drugs are required to deliver the correct treatment to the right patient at the right time [68]. Therefore, there is a long way to go in treating cancer patients.
